# Cabinet of Entomology for Sale

**Published:** 1833

**Authors:** 


### VI. Cabinet of Entomology for Sale.

Mr. N. M. Hentz, late Professor of Natural History in the University of North Carolina, but now resident in this city, a diligent and learned Entomologist, offers for sale his extensive

Cabinet of American Insects. It embraces 30,000 specimens, the collections of many years of laborious research, directed chiefly upon the great order Coleoptera. It is in a state of excellent preservation, and would be sold cheap, as the Professor has turned his attention to other pursuits.

